# The work–recovery cycle of kidney strain and inflammation in sugarcane workers following repeat heat exposure at work and at home

**DOI:** 10.1007/s00421-024-05610-3

**Published:** 2024-10-05

**Authors:** Rebekah A. I. Lucas, Erik Hansson, Bethany D. Skinner, Esteban Arias-Monge, Catharina Wesseling, Ulf Ekström, Ilana Weiss, Zoey E. Castellón, Scarlette Poveda, Fatima I. Cerda-Granados, William Jose Martinez-Cuadra, Jason Glaser, David H. Wegman, Kristina Jakobsson

**Affiliations:** 1 School of Sport, Exercise and Rehabilitation Sciences, University of Birmingham, Edgbaston, Birmingham, B15 2TT UK; 2 La Isla Network, Washington, DC USA; 3 School of Public Health and Community Medicine, Sahlgrenska Academy, University of Gothenburg, Gothenburg, Sweden; 4 Unit of Occupational Medicine, Institute of Environmental Medicine, Karolinska Institutet, Stockholm, Sweden; 5 Department of Laboratory Medicine, Lund University, Lund, Sweden; 6 University of Massachusetts Lowell, Lowell, MA USA; 7 Department of Occupational and Environmental Medicine, Sahlgrenska University Hospital, Gothenburg, Sweden

**Keywords:** Recovery, Kidney strain, Inflammation, Heat stress, Worker health

## Abstract

**Purpose:**

To examine heat exposure at work and home and the work–recovery cycle and temporal variation of kidney strain, muscle injury and inflammation biomarkers in sugarcane workers.

**Methods:**

20 male sugarcane workers (age: 33 ± 7 years) with a workplace Rest.Shade.Hydration (RSH) intervention were observed over 4 days, at the end (18 h post-shift recovery) and beginning of a work week (42 h post-shift recovery). Measures included work intensity (heart rate), gastro-intestinal temperature, estimated body core temperature (using heart rate), fluid consumption, pre- and post-work blood and urine samples, physical activity (accelerometery) away from work, plus ambient heat exposure at work and home.

**Results:**

On workdays, workers awakened at approx. 02:40 after 5 h sleep in ~ 30 °C. Across work shifts, daily average WBGT ranged from 26 to 29 °C (cooler than normal) and average workload intensity ranged from 55 to 58%HR_max_. Workers reported consuming ~ 8 L of water and ~ 4 × 300 mL bags of electrolyte fluid each day. Serum creatinine, cystatin C and creatine phosphokinase markedly increased post-work and decreased during recovery; serum potassium did the opposite (all *p* < 0.01). Biomarker concentration changes were similar between recovery periods (18 h vs. 42 h; all *p* > 0.27). C-reactive protein was the highest at the end of the work week (*p* = 0.01).

**Conclusion:**

Despite RSH intervention, cross-shift kidney strain was marked (recovering overnight) and systemic inflammation increased over the work week. Thus, biomonitoring of kidney function in occupational populations should be performed before a work shift at any point in the work week. This is essential knowledge for field studies and surveillance.

**Supplementary Information:**

The online version contains supplementary material available at 10.1007/s00421-024-05610-3.

## Introduction

In recent decades, outbreaks of chronic kidney disease of non-traditional origin (CKDnt) have emerged globally at epidemic rates in Mesoamerica, Sri Lanka, India, and other regions (Wesseling et al. 2020; Glaser et al. 2016). In Mesoamerica, CKDnt has been characterized as an occupational disease driven by heat stress from strenuous manual labor performed in hot environments (Wesseling et al. 2020). Subsequently, it has been postulated that episodes of acute kidney injury (AKI) or kidney strain, due to repeat occupational heat exposure, cause chronic functional and structural kidney damage that develops into CKDnt (Hansson et al. 2020; Chawla and Kimmel 2012). Indeed, workforces at high risk of developing CKDnt show a decline in kidney function across work shifts (Butler-Dawson et al. 2019; Santos et al. 2015; García-Trabanino et al. 2015; Wesseling et al. 2016; Sorensen et al. 2019) and across harvest seasons (Kupferman et al. 2018; Hansson et al. 2019b, 2020; Wesseling et al. 2016). Furthermore, cross-shift changes in kidney function (as measured via serum creatinine) over a week have been shown to forecast end-of-harvest kidney function in sugarcane workers (Dally et al. 2020). Thus, following an acute insult, insufficient restoration of the kidney to normal morphology and function may lead to permanent damage and CKD (Lameire et al. 2013). Added to this, C-reactive protein (CRP, a sensitive biomarker of inflammation) has been shown to increase among sugarcane workers performing the physically most demanding tasks during the harvest season, especially in those developing kidney injury (Hansson et al. 2020, 2019a). Whether CRP dynamics across the working week signal an occupation-induced pro-inflammatory state in sugarcane workers has not been previously explored.

The importance of recovery in the possible pathogenesis of CKDnt has been outlined by Hansson et al. (2020) and shares many parallels with the ‘overtraining syndrome’ in athletes, where a maladaptive inflammatory response develops due to frequent exposure to exercise-induced physiological strain and insufficient recovery (Kreher and Schwartz 2012; Armstrong et al. 2022). Notably, heat stroke/illness can also trigger the overtraining syndrome (Kreher and Schwartz 2012). The nature of industrial agricultural sugarcane work in Mesoamerica is that it is repetitive and physically very strenuous (Lucas et al. 2022, 2015). During a harvest season, workers can work between 5 to 11 h/day and 6 to 7 days a week, for 5 to 6 months. Thus, workers’ opportunity for recuperative rest is limited and likely insufficient. Added to this, organizational factors such as the piece rate payment system, used throughout industrial agriculture, incentivizes workers to work harder, inhibiting their opportunity or willingness to take rest breaks and recover during work shifts (Mitchell et al. 2018; Spector et al. 2015). Additionally, workers often have other tasks they undertake after a work shift, such as subsistence farming or a second job (Raines et al. 2014), which further limits their rest and recovery. Workers’ physical activity, sleep patterns and ambient living temperatures away from work have not been described. Yet, recovery may be a crucial factor in the development of CKDnt among agricultural workers.

**Fig. 1:**
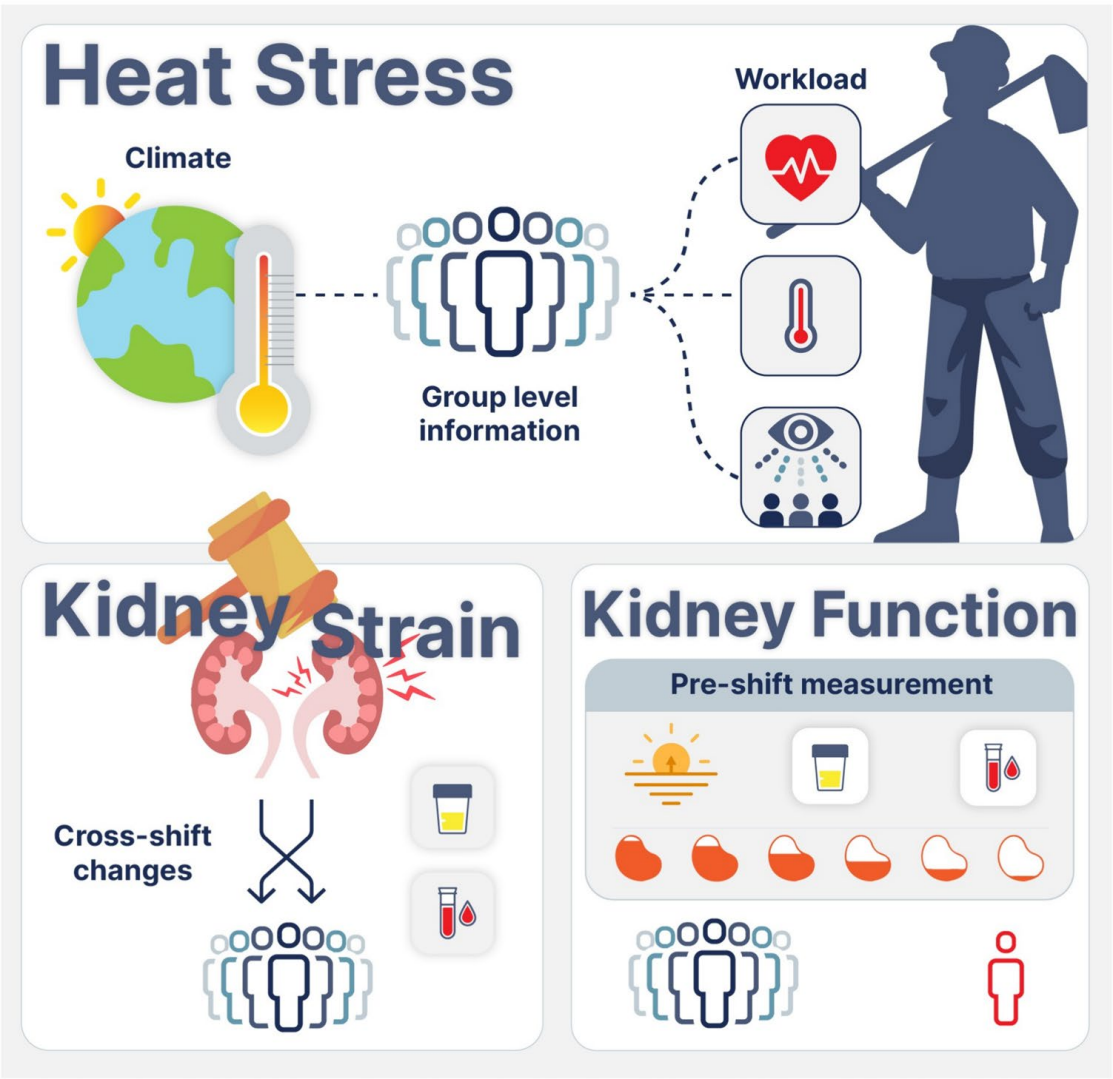
A conceptual framework for assessing and reporting data from health surveillance and cross-sectional field-based investigations in occupational settings. Climate and workload measurements indicates workers’ heat stress exposure at a group level. Cross-shift changes in biomarkers (i.e., pre- to post-work shift) indicates the degree of physiological strain the kidney undergoes and can be used for surveillance at a group level. Kidney function measures indicate the kidney’s capacity to maintain homeostasis and therefore kidney health, injury (e.g., AKI) or disease (e.g., CKD) as defined by clinical criteria. Kidney function measures can describe kidney health at a group or individual level

The aim of the present study was to assess cross-shift changes and post-shift recovery of kidney function and muscle injury biomarkers, assess the inflammatory response to a working week, and to characterize the work and home environment (including workers’ physical activity, sleep patterns and ambient living temperatures away from work) to understand the context of this work–recovery cycle. Therefore, the recovery of kidney function and muscle injury biomarkers after one night’s recovery from a work shift (at the end of a working week), compared to two nights and one day’s recovery (at the beginning of a working week, after a day off) was explored. Moreover, a better understanding of the short-term temporal variation in biomarkers of kidney function (i.e., kidney strain) is also needed to inform field study and health surveillance protocols in occupational settings (Fig. 1).

## Methods

### Setting and study population

The current study was conducted as part of the Adelante Initiative, a program aimed to assess the impact of evidence-based recommendations to improve work conditions and reduce kidney dysfunction among sugarcane workers at the sugarcane mill Ingenio San Antonio (ISA) in Chinandega, Nicaragua (https://adelanteinitiative.org/). The Adelante Initiative has now been systematized in the transdisciplinary Prevention, Resilience, Efficiency and Protection (PREP) research program (https://www.enbel-project.eu/projects-page/prep).

For this observational study, 20 male burned cane-cutting workers were opportunistically recruited from one randomly selected *cuadrilla* (working group) of approximately 50–60 persons. These workers were also participating in a larger longitudinal study (Hansson et al. 2019a). Burned cane cutters were recruited for this study as this job is the most strenuous and difficult (Lucas et al. 2022). No females at ISA are employed as burned cane cutters and therefore could not be included in the participant sample. Data collection for the present study occurred during the 2020 mid-harvest season, over the course of four days: Friday 21st to Monday 24th February. Friday, Saturday, and Monday were workdays, while Sunday was a rest day. During the current study, ISA had an enhanced Rest.Shade.Hydration intervention for burned cane cutters, with a maximum six-hour workday, hourly mandated rest breaks in the shade, easy access to cool water plus electrolyte solutions (300 mL *bolis*/bags; sugar 7%, 150 mg sodium chloride and 60 mg potassium phosphate), with lunch provided at the end of the workday. Further aspects of the enhanced intervention implementation during the 2020–2021 harvest are detailed in Glaser et al. (2022). Ethical approval for this study was given by the Comité de Ética para Investigaciones Biomédicas (CEIB), Facultad de Ciencias Médicas, Universidad Nacional Autónoma de Nicaragua (UNAN—León (FWA00004523/IRB00003342).

### Protocol

#### Work shift context

Climate measures were recorded in the field alongside workers across their workday via portable weather stations (QuesTemp 34, 3 M). After arriving in the field at dawn (approx. 05:30) via ISA-provided bus transportation, the 20 workers gave a blood and spot urine sample (pre-shift) before being equipped with a heart rate (HR) monitor. A subset of nine workers on Friday and a different nine workers on Monday volunteered to swallow a calibrated gastro-intestinal temperature (T_GI_) telemetry pill ~ 1 h prior to starting work. The work shift (approx. 6 h long) then commenced. During work shifts, qualitative work observations were performed by occupational hygienists and senior occupational physicians. Workers’ fluid consumption across their shift was also monitored hourly by ISA field personnel. Upon finishing work at approximately noon, workers gave a blood and urine sample (post-shift) and completed a brief questionnaire wherein they were asked to rate how strenuous they felt their workday was.

#### Context away from work (i.e., during post-work recovery)

At the end of their work shift, workers were given a wrist-based accelerometer and HR monitor and a lanyard with a key fob and an iButton temperature sensor attached. Workers were asked to wear the accelerometer and HR monitor continuously and to keep the key fob near their person (uncovered, within ten meters, or in their bedroom when sleeping) until the start of their next work shift. From these measures, each participant’s physical activity and local ambient air temperature away from work were assessed from Friday post-shift till Saturday pre-shift (18 h), then again from Saturday post-shift till Monday pre-shift (42 h; including one rest day, Sunday).

### Measures

#### During the work shift

To assess workers’ individual work intensity, HR was continuously recorded (0.1 s intervals) from a sensor fitted on a chest strap (Polar Team Pro Sensor, Polar Electro, Kempele, Finland). Heart rate data are expressed as percentage of maximal HR (%HR_max_), with a regression equation used to predict HR_max_ (208—0.7 × age) (Tanaka et al. 2001). Workload was categorized based on %HR_max_ as: Maximal (91–100%); Very Hard (81–90%); Hard (71–80%); Moderate (61–70%); Light Moderate (51–60%); Light (≤ 50%).

Average and maximal T_GI_ across the work shift were calculated to assess workers’ internal body temperature and level of heat strain. Gastrointestinal temperature (eCelsius Performance capsule; BodyCap medical) was measured (30 s intervals) in a subset of 18 workers due to a limited number of T_GI_ receivers (e-Viewer, BodyCap, Caen, France).

Blood and spot urine samples were taken in the field at five time points across the four-day protocol (Friday post-shift, Saturday pre- and post-shift, and Monday pre- and post-shift). In the field, urine samples were analyzed using dipstick urinalysis for specific gravity, pH, leukocytes, and blood (Multistix Reagent Strips, Siemens AG, Munich, Germany) with an automated reader. Serum samples were separated at a laboratory in ISA. Serum and urine samples were then frozen (− 77 °C) before being transported to Sweden. Blood serum samples (10–20 mL at each time point) were analyzed (for creatinine, cystatin C, C-reactive protein (CRP), creatine phosphokinase (CPK), sodium, potassium, urea, and uric acid) at Skåne University Hospital in Lund, using a Cobas 701 instrument (Roche Diagnostics, Basel, Switzerland). Urine samples were analyzed (for albumin and creatinine) using a Cobas instrument at Sahlgrenska University Hospital, Gothenburg, Sweden.

#### Context away from work

Physical activity away from work was tracked using a wrist-based accelerometer monitor (Polar A370, Polar Electro, Kempele, Finland). Time spent lying, sitting, standing, walking, and exercising plus total sleep duration, wake time and bedtime were exported using Polar FlowSync software and extracted from a web platform (Polar Flow, Polar Electro). Time spent at each activity level was converted to a percentage of the total time the accelerometer was worn on each day (from midnight to midnight). This was to account for the differing total length of time the accelerometer was worn on different days, thus Friday approx. 12:00–00:00, ~ 11–12 h; Saturday approx. 00:00–06:00 and 12:00–00:00, ~ 17–18 h excluding the work shift; and Sunday 00:00–00:00, ~ 24 h.

Away from work, ambient air temperature (equivalent to dry bulb air temperature) was measured at 5-min intervals using iButton temperature sensors (DS-1921 H model, Maxim Integrated, San Jose, USA), attached to key fobs (IMAC electronic solutions, UK).

### Data analysis

Outliers in heart rate data (e.g., > 220 beats/min) and T_GI_ data (≥ 5 °C difference between adjacent data points; Hertzberg et al. 2017) were removed.

Work/home context data were analyzed using one-way repeated measures ANOVAs or mixed-effects model (factor: time; three levels: Friday, Saturday, Monday) when appropriate, to compare measures of workload intensity, physical activity away from work, sleep patterns and environmental heat exposure. Differences in T_GI_ between work shifts were analyzed using an unpaired *t*-test. Post hoc analyses were performed using Tukey’s HSD test and statistical significance was defined as *p* < 0.05. Work/home context data are presented as mean values and 95% confidence intervals [CI] unless otherwise stated.

#### Work–recovery cycle: kidney, hydration and muscle damage biomarker dynamics

Changes in kidney glomerular filtration within individuals during the study period were modeled as the fold changes in concentrations of the biomarkers creatinine and cystatin C. As these biomarker concentrations could not be assumed to be in a steady state during the short time frame of the current study, changes in estimated glomerular filtration rate (eGFR) have not been reported herein as interpreting creatinine or cystatin C changes concentrations as eGFR changes would be misleading (Christiadi et al. 2021). However, in the supplementary material, we have reported CKD-EPI eGFR (which simply can be seen as a normalization with respect to age, but not representing true eGFR) to allow comparison with other studies reporting non-steady-state changes in eGFR.

For serum data, six probable errors among the 8 compounds analyzed in 100 samples (< 0.01%) were excluded from analysis. For creatinine, one value was excluded as it implied a physiologically impossible rapid increase in serum creatinine (from 0.83 to 1.89 mg/dL in 6 h). Three cystatin C results (0.42–0.57 mg/L) were excluded as they were well below the reference interval (0.75–1.06 mg/L; Fig. 4 A) and implied very rapid changes within workers. None of the excluded results corresponded to parallel cystatin C or creatinine values at the same time point. One urea concentration and one uric acid concentration were removed as they were > 3 standard deviations away from the remainder of the material and implied very rapid changes within workers, deemed unlikely. One individual had a large, but plausible, increase in CPK across the weekend, and models were run with and without this individual.

Concentration change ratios of kidney strain biomarkers (serum cystatin C, creatinine and urea; and urine albumin), muscle injury (CPK), hydration status (serum albumin), electrolyte balance (serum potassium and sodium) and purine metabolism (serum uric acid) were modeled using three different linear mixed models. Main cross-shift versus recovery changes were assessed by including only one fixed effect, two-level variable for recovery (change post-to-pre) and cross-shift (change pre-to-post) periods. Differences in concentration changes during recovery following 18 h (Friday afternoon to Saturday morning) or 42 h (Saturday afternoon to Monday morning) recovery were assessed by additionally including a two-level variable for 18 or 42 h recovery periods. Differences in concentration changes during the work shift following 18 or 42 h recovery were assessed in models including the initial recovery/cross-shift variable and a two-level variable for 18 or 42 h recovery periods preceding the work shift. A random intercept for each individual was included in all models.

Model assumptions of normality and homoscedasticity of residuals were checked using graphical methods and found to be satisfactory for all biomarkers and models, except for one individual having a large but plausible increase in CPK during the weekend rest.

#### Accumulated working week inflammatory response

The inflammatory response during the work week was assessed by modeling log-transformed serum CRP levels as a function of pre- or post-shift and pre- and post-weekend (Saturday versus Monday) measurement occasion. Mixed effects tobit regression was used as CRP concentrations below 0.6 mg/L were not quantified. Changes in CRP levels across work shifts or nights were not assessed as the time frame was considered too short for this marker to respond (Schmit and Vincent 2008).

Biomarker concentrations and concentration fold changes are reported as medians and IQRs.

## Results

The workers who participated were (mean ± SD) 33 ± 7 years, with weight 68.3 ± 11.2 kg, and height 164 ± 5 cm. Their eGFR using serum creatinine was 115 (IQR, 90–123) mL/min/1.73m^2^ (calculated from Monday morning sample at beginning of work week using the CKD-EPI formula (Levey et al. 2009)).

### Context during and away from work

**Fig. 2:**
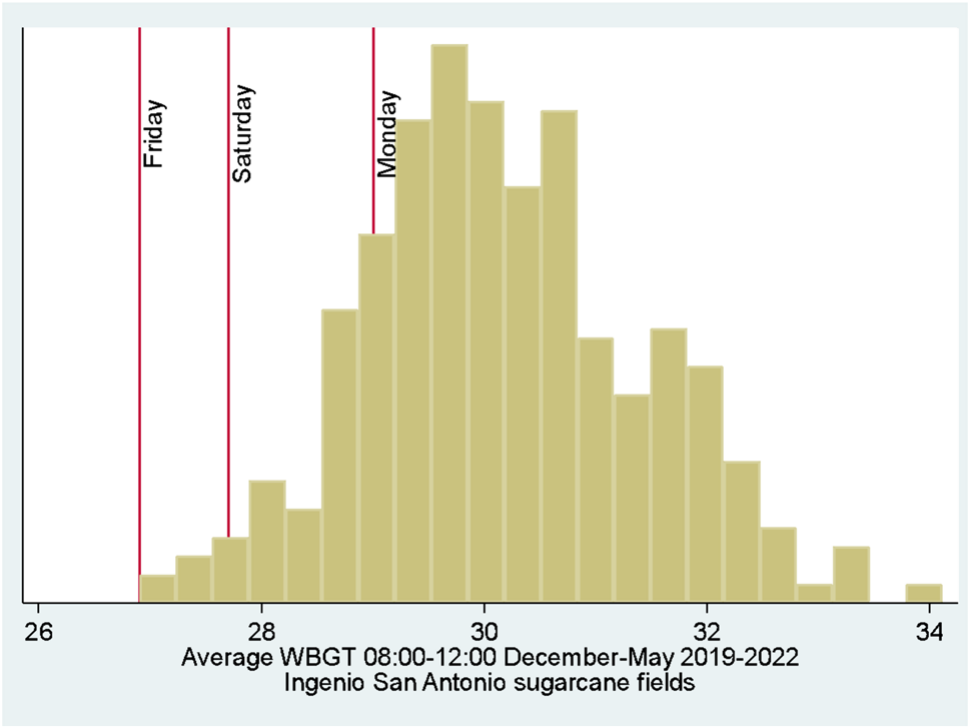
Daily average Wet Bulb Globe Temperature (WBGT) recorded in the field alongside workers during work shifts (from 08:30 to 12:00) across harvests (December–May, 2019–2022). For the current study, daily average WBGT during data collection days are indicated by red lines

*Heat stress exposure during work:* Daily average WBGT (08:30–12:00) was higher on Monday compared to both Friday and Saturday though lower across all assessment days than most days experienced during sugarcane harvest seasons (Fig. 2). Daily average WBGT was similar between Friday and Saturday (*p* = 0.18). WBGT reached a maximum of 27.7 °C, 29.3 °C, and 30.8 °C for Friday, Saturday and Monday, respectively. Daily ambient air temperatures in the field reached maximum values of 34.5 °C (Friday), 36.3 °C (Saturday), and 33.6 °C (Monday), with no difference in daily average air temperatures across workdays. Daily average and maximium WBGT, dry bulb temperature, globe temperature and relative humidity for each work shift are reported in the supplementary material.

*Workload:* Work shift duration was approx. 5 h, slightly longer on Friday, when compared to Saturday (*p* < 0.01) or Monday (*p* < 0.01; Table 1) but of similar duration for Saturday and Monday (*p* = 0.66). Average workload intensity (as indexed by %HR_max_) across a work shift (inclusive of work and rest periods) was somewhat higher on Monday when compared to Saturday (*p* < 0.01) but similar to Friday (*p* = 0.11; Table 1). Workload intensity was similar between Friday and Saturday (*p* = 0.15). Notably, on Monday, workers spent a greater proportion of their work shift working ‘hard’ (71–80%HR_max_) when compared to Saturday (*p* = 0.03; Table 1).

**Table 1:** Work shift duration, daily average workload intensity (as indexed by %HR_max_) plus time (min; shaded rows) and percentage of work shift spent at different workload intensities across three workdays (inclusive of work and rest periods) for burned cane-cutting workers (*n* = 20)

	Friday	Saturday	Monday
Work shift duration (hrs)	05:13 [05:00, 05:26]	04:26 [04:08, 04:43]*	04:33 [04:18, 04:47]*
Average workload intensity (%HR_max_)	57% [55, 59]	55% [53, 57]	58% [56, 61]#
Workload intensity categories			
Light (< 50%HR_max_)	25% [18, 32]	34% [31, 37]	26% [21, 31]#
	91 min [59, 123]	110 min [98, 121]	84 min [68, 100]
Light–moderate (51–60%HR_max_)	29% [22, 35]	28% [22, 34]	24% [19, 29]
	104 min [77, 132]	91 min [69, 114]	78 min [62, 94]
Moderate (61–70%HR_max_)	30% [22, 38]	31% [24, 38]	32 [25, 39]
	104 min [74, 134]	98 min [75, 121]	105 min [81, 129]
Hard (71–80%HR_max_)	14% [8, 20]	7% [3, 12]	15% [10, 21]#
	46 min [27, 65]#	23 min [9, 36]	49 min [31, 67]#
Very hard (81–90%HR_max_)	2% [− 1, 6]	0% [0, 0]	3% [− 1, 6]
	7 min [− 3, 17]	1 min [0, 2]	8 min [− 2, 18]
Maximal (91–100%HR_max_)	0% [0, 0]	0% [0, 0]	0% [0, 0]
	0 min [0, 0]	0 min [0, 0]	0 min [0,0]

Data presented as mean [95% confidence intervals]

^*^Significantly different to Friday

^#^Significantly different to Saturday

*Heat strain and hydration during work:* Maximum T_GI_ was on average 0.3 °C [0.0, 0.6] higher during Monday work shift compared to Friday (37.8 [37.6, 38.0] vs. 37.5 [37.3, 37.7] °C, *p* = 0.02). Average T_GI_ across a work shift was higher on Monday (37.1 °C [36.8, 37.3]) when compared to Friday (36.7 °C [36.4, 37.0]; *p* = 0.05). However, T_GI_ was greatly affected by water intake in some workers, resulting in substantial troughs in T_GI_. On average, registered water intake was similar during Friday, Saturday and Monday work shifts (7.9 L [7.2, 8.8], 8.2 L [7.1, 9.3], and 7.6 L [6.9, 8.3], respectively; *p* = 0.62). Similarly, workers consumed the same number of *bolis* (300 mL bags of electrolyte fluid) during Friday, Saturday and Monday work shifts (4 bolis [4, 5], 4 bolis [3, 4], and 4 bolis [3, 5], respectively; *p* = 0.64).

*Physical activity and sleep patterns away from work:* On their rest day (Sunday; midnight to midnight), workers spent the majority of their classified time lying (8:43 h), and less but similar amounts of time sitting (5:58 h) and standing (6:21 h; Table 2). They spent the least amount of time on their rest day walking (2:14 h) and exercising (0:15 h; Table 2). On a workday, workers woke at approximately 02:40, compared to approximately 05:20 on their day off. Prior to a workday, workers went to bed at approximately 21:30, compared to 23:00 prior to a rest day. On average, workers slept the most on Saturday night, prior to their rest day (Table 2).

**Table 2:** Burned cane-cutting workers (*n* = 20) physical activity and sleep patterns after two work shifts (Friday and Saturday) and during one rest day (Sunday)

	Friday	Saturday	Sunday
Lying (h:min)	03:03 [02:29, 03:43]	04:50 [04:10, 05:25]*	08:43 [07:44, 09:42]*^#^
	26% [21, 31]	27% [24, 30]	37% [32, 43]*^#^
Sitting (h:min)	02:32 [02:06, 02:58]	04:44 [04:04, 05:24]*	5:58 [05:01, 06:53]*^#^
	22% [18, 25]	27% [23, 30]	25% [21, 29]
Standing (h:min)	03:02 [02:39, 03:24]	05:40 [04:57, 06:23]*	6:21 [05:30, 07:13]*
	26% [23, 29]	32% [28, 36]*	27% [23, 30]
Walking (h:min)	2:21 [01:50, 02:52]	2:03 [01:22, 02:44]	2:14 [01:28, 03:02]
	20% [16, 25]	11% [8, 15]*	9% [6, 13]*
Exercising (h:min)	00:47 [00:26, 01:08]	00:33 [00:20, 00:46]	00:15 [00:03, 00:27]
	7% [4, 10]	3% [2, 4]	1% [1, 2]*^#^
Sleep Duration (h:min)	^‡^05:14 [04:42, 05:47]	^‡^06:27 [05:11, 07:42]	04:57 [04:26, 05:28]
Bedtime (24 h time)	^‡^21:32 [20:56, 22:09]	^‡^22:56 [21:53, 00:00]	21:38 [21:03, 22:14]^#^

Shaded rows indicate the percentage of time spent performing each activity. Duration of monitoring: Friday—12:00–23:59; Saturday—00:00–06:00, 12:00–23:59 (excluding work shift); Sunday—00:00–23:59. Data presented as mean [95% confidence intervals]. *n* = 18 for sleep pattern data on Friday and Saturday

^‡^Two workers removed the accelerometer at night

^*^Significantly different to Friday

^#^Significantly different to Saturday

**Fig. 3:**
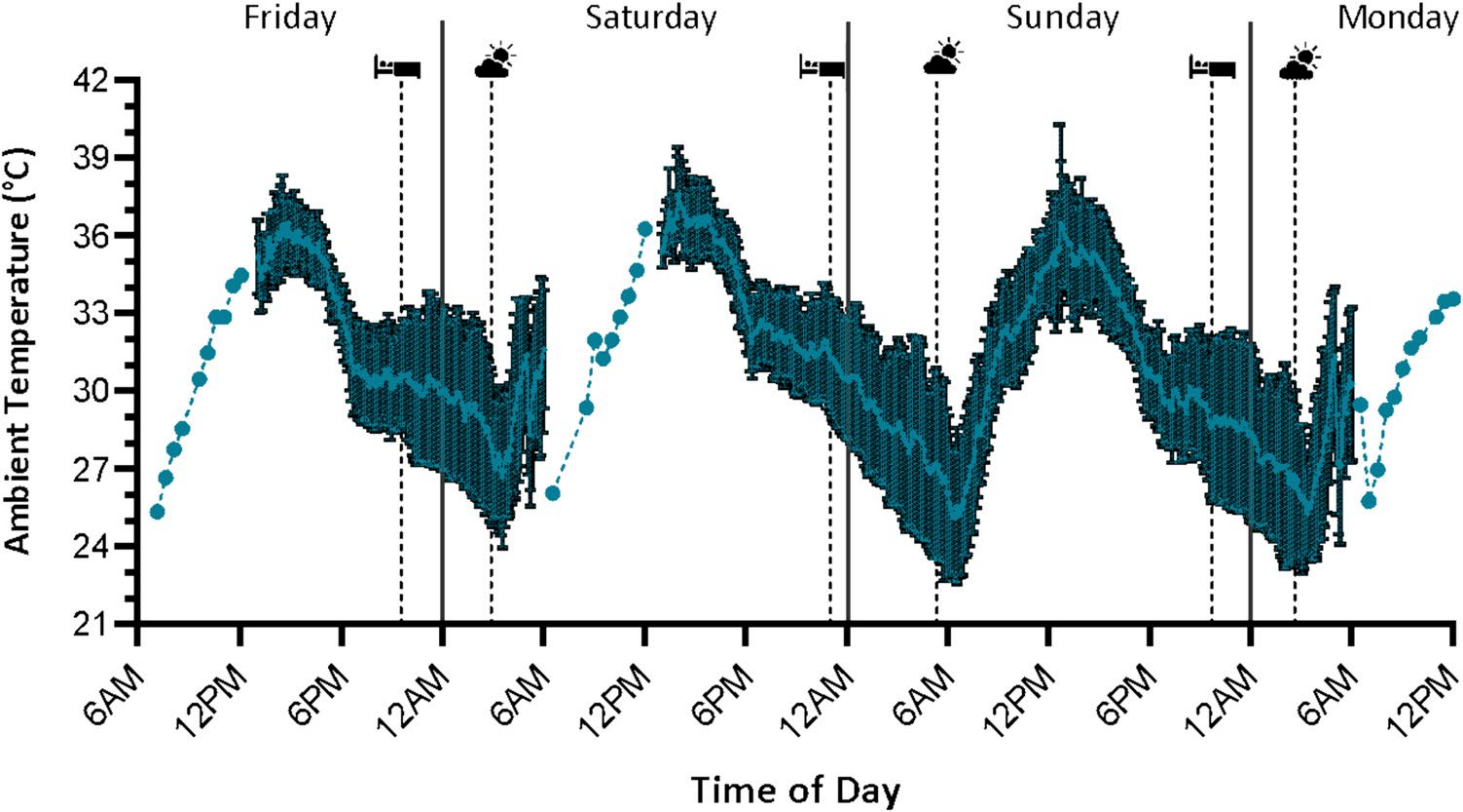
Mean ambient temperature across three workdays and one recovery day. Air temperature (or dry bulb air temperature) during the work shift (~ 6am–12 pm) was measured alongside workers (burned cane cutters) using a portable weather station (QuesTemp 34), while average ambient temperature away from work was measured using iButton temperature sensors workers kept near their person (*n* = 20). Dashed lines indicate mean bedtime and wake time (based on data from wrist-based accelerometer and HR monitors). Solid lines indicate the start/end of each day

*Ambient air temperature away from work:* Ambient air temperature away from work is displayed in Fig. 3. When sleeping, ambient temperature was on average 29.8 °C, 29.1 °C, and 28.0 °C during Friday, Saturday and Sunday nights, respectively, with air temperatures when away from work ranging from 22.6 to 36.5 °C.

### Kidney, hydration, muscle damage and inflammation biomarker dynamics

**Fig. 4:**
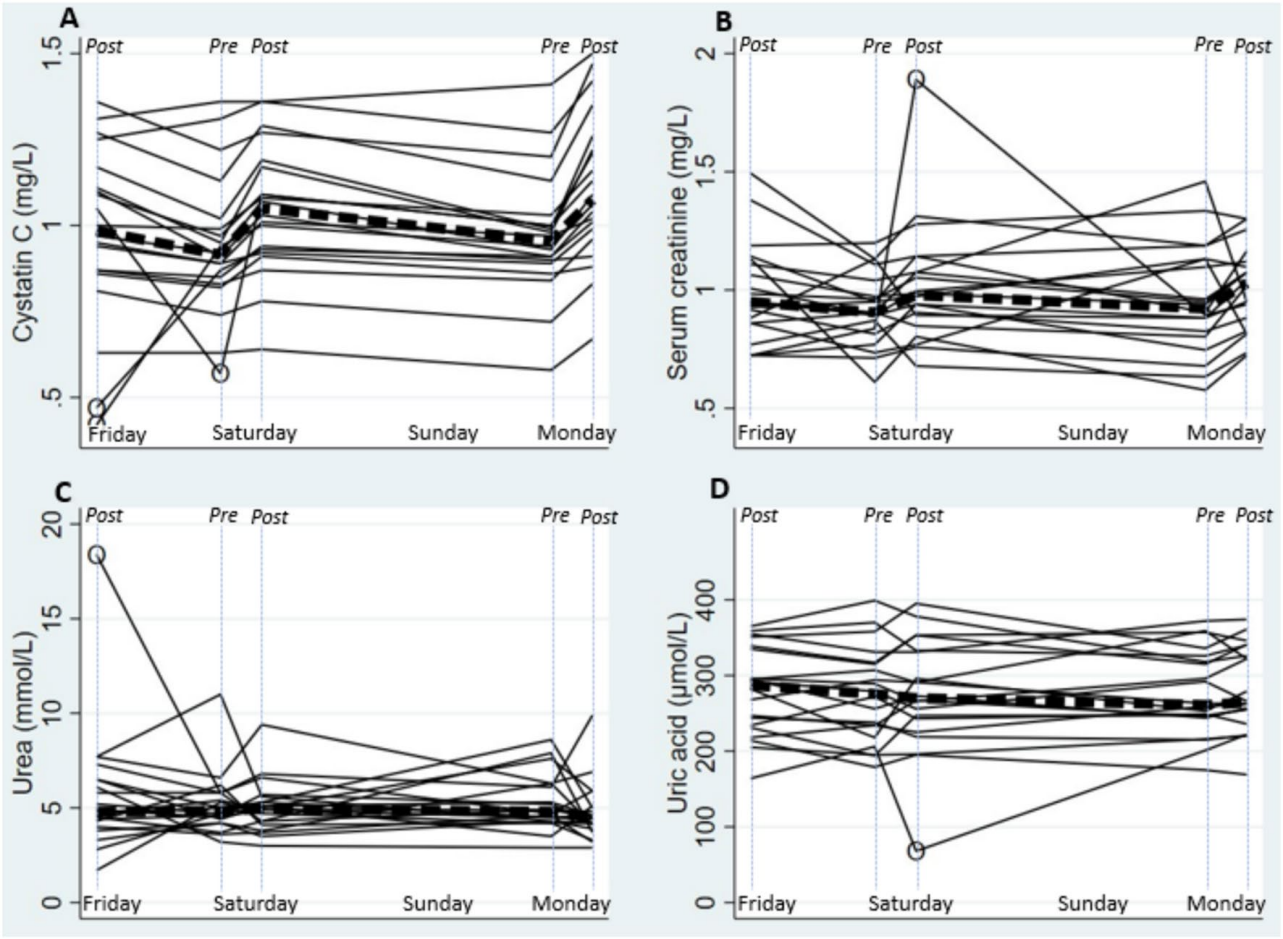
Changes in serum cystatin C (Panel A), serum creatinine (Panel B), serum urea (Panel C), and serum uric acid (Panel D) across the study period: Friday (post-shift), Saturday (pre- and post-shift), and Monday (pre- and post-shift). Each solid line represents a burned cane cutter (*n* = 20). Encircled (O) measurement points were excluded from regression model analyses. Dashed lines indicate reference value at Lund University hospital laboratory

**Fig. 5:**
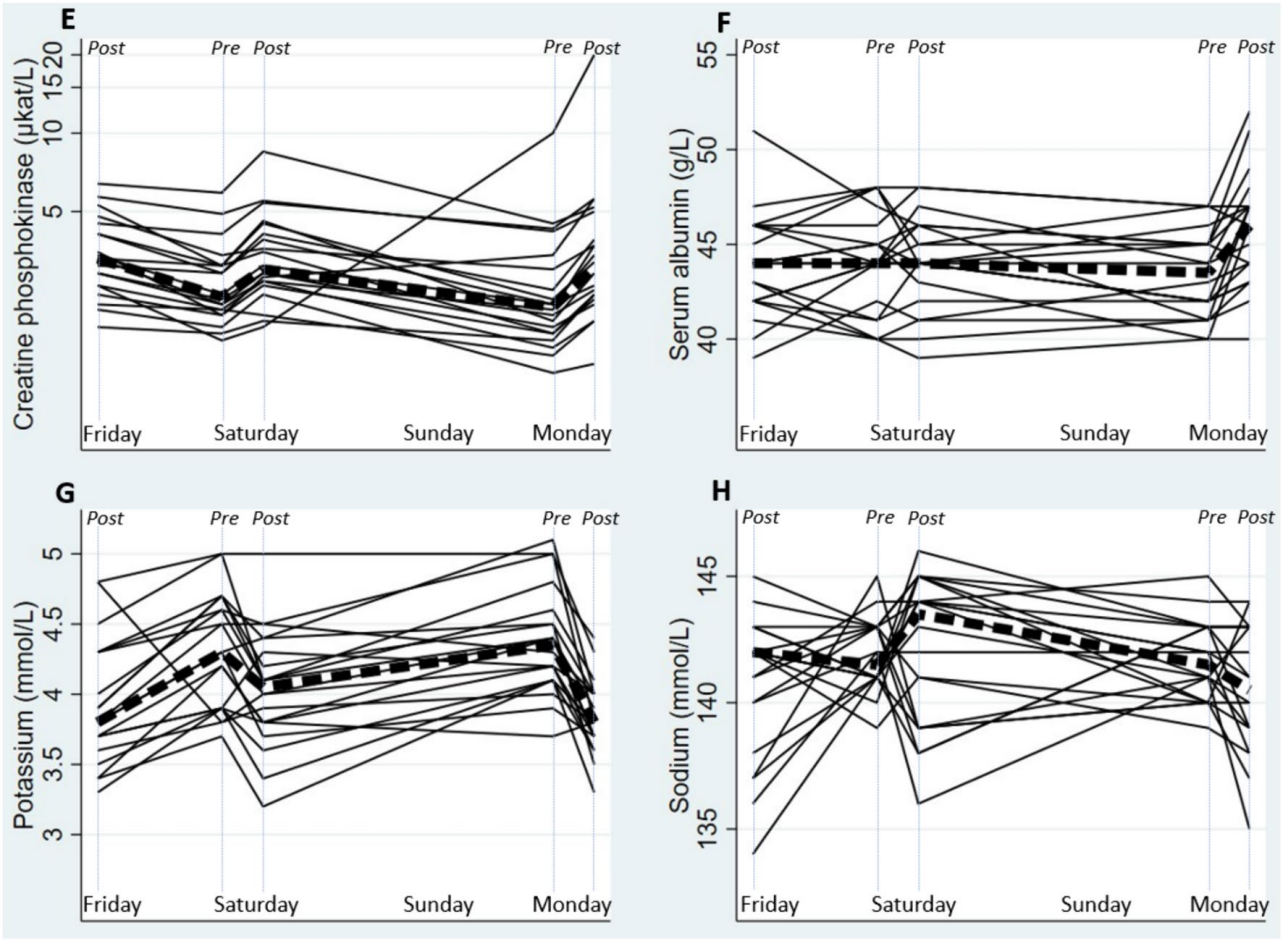
Changes in serum creatine phosphokinase (Panel E), serum albumin (Panel F), serum potassium (Panel G), and serum sodium (Panel H) across the study period: Friday (post-shift), Saturday (pre- and post-shift), and Monday (pre- and post-shift). Each solid line represents a burned cane cutter (*n* = 20). Dashed lines indicate reference value at Lund University hospital laboratory

*Work–recovery cycle:* Serum creatinine, cystatin C, CPK and albumin all increased during work shifts and decreased during recovery periods (*p* < 0.01), whereas serum potassium showed an opposite pattern (*p* < 0.01) and there was no clear work–recovery dynamic for serum uric acid, urea or sodium (Fig. 4, Table 3). Cross-shift changes were similar between Monday and Saturday work shifts for all markers except albumin (*p* < 0.01) and cystatin C (*p* = 0.02), which showed greater increases during Monday work shift. There was no difference in recovery changes between Friday and Saturday or Saturday and Monday recovery periods for any marker, except for CPK. After excluding one individual with a large CPK increase during the weekend, there was a larger CPK decrease during the longer rest (*p* = 0.01). Urine albumin levels were uniformly undetectable or very low (Fig. 5).

**Table 3:** Kidney, hydration, inflammation and muscle damage biomarker concentrations and concentration fold changes post-shift to pre-shift after one night’s recovery (Friday post-shift to Saturday pre-shift, 18 h off work) and two night’s recovery (Saturday post-shift to Monday pre-shift, 42 h off work)

Biomarker	Concentrations, median (IQR)					Concentration fold changes, median (IQR)				*p*-values		
	Friday PM	Saturday AM	Saturday PM	Monday AM	Monday PM	Friday–Saturday recovery (18 h)	Saturday cross-shift (post 18 h recovery)	Saturday–Monday recovery (42 h)	Monday cross-shift (post 42 h recovery)	Cross-shift v.s. recovery effect	Different recovery effect 18 h vs 42 h	Different cross-shift effect after 18 h vs. 42 h recovery
Serum cystatin C (mg/L)	0.99	0.92	1.05	0.96	1.08	0.95	1.09	0.94	1.14	< 0.01	0.63	0.02
	(0.87–1.14)	(0.84–1.01)	(0.93–1.18)	(0.90–1.02)	(0.98–1.24)	(0.89–0.98)	(1.04–1.13)	(0.89–0.96)	(1.11–1.18)			
Serum creatinine (µmol/L)	0.95	0.90	0.98	0.92	1.03	0.95	1.06	0.94	1.10	< 0.01	0.45	0.82
	(0.86–1.12)	(0.83–1.01)	(0.87–1.11)	(0.81–1.13)	(0.85–1.11)	(0.87–1.04)	(1.02–1.16)	(0.90–1.04)	(1.04–1.17)			
Serum Uric acid (µmol/L)	287	276	270	261	265	0.97	1.00	0.98	1.03	0.12	0.76	0.65
	(233–337)	(226–316)	(234–332)	(245–322)	(246–333)	(0.93–1.06)	(0.92–1.11)	(0.89–1.11)	(0.98–1.06)			
Serum Urea (mmol/L)	4.8	4.9	5.0	4.8	4.5	1.00	1.04	0.98	0.95	0.10	0.66	0.59
	(4.2–6.5)	(4.3–5.8)	(4.2–5.5)	(4.3–6.2)	(4.0–5.1)	(0.82–1.27)	(0.90–1.15)	(0.87–1.24)	(0.78–1.01)			
Urine albumin	–	–	< 2 (< 2– < 2)	< 2 (< 2– < 2)	< 2 (< 2–4.0)	–	–	–	–	–	–	–
Albumin (g/L)	44	44	44	43.5	46	1.00	1.00	0.99	1.03	< 0.01	0.27	< 0.01
	(42–46)	(41.5–45.5)	(42.5–46)	(41.5–45)	(43.5–47)	(0.97–1.05)	(0.98–1.02)	(0.95–1.02)	(1.02–1.10)			
Urine density	1015 (1010–1020)	1015 (1015–1020)	1015 (1013–1018)	1010 (1010–1015)	1015 (1010–1018)	–	–	–	–	–	–	–
Urine pH	6 (6–7)	6.5 (6–6.8)	6.5 (6–7)	6.8 (6–7)	6 (6–7)	–	–	–	–	–	–	–
Urine creatinine (mmol/L)	–	–	2.8 (1.3–5.6)	2.3 (1.3–3.6)	3.6 (1.1–7.1)	–	–	–	–	–	–	–
Serum CPK (µkat/L)	3.3	2.4	3.0	2.2	3.1	0.80	1.30	0.67	1.36	< 0.01	0.48	0.46
	(2.6–4.3)	(2.1–3.1)	(2.7–4.3)	(1.7–3.2)	(2.4–4.5)	(0.71–0.92)	(1.13–1.37)	(0.54–0.79)	(1.23–1.54)			
Serum CRP (mg/L)	0.7	1.3	1.3	1.1	LOQ	–	–	–	–	–	–	–
	(LOQ-1.8)	(LOQ-2.3)	(LOQ-2.1)	(LOQ-1.4)	(LOQ-1.4)							
Serum sodium (mmol/L)	142	142	144	142	141	1.00	1.01	0.99	0.99	0.22	0.08	0.19
	(139–143)	(141–143)	(139–145)	(140–143)	(139–143)	(0.99–1.02)	(0.98–1.02)	(0.99–1.01)	(0.99–1.00)			
Serum potassium (mmol/L)	3.8	4.3	4.1	4.4	3.8	1.11	0.91	1.09	0.88	< 0.01	0.53	0.02
	(3.7–4.3)	(3.9–4.7)	(3.8–4.4)	(4.1–4.6)	(3.7–4.0)	(1.05–1.17)	(0.88–1.00)	(1.02–1.15)	(0.84–0.93)			

*CPK* creatine phosphokinase, *CRP* C-reactive protein

*Accumulated working week response:* Although CRP levels overall were mostly low, there was a tendency toward CRP being higher at the end of the working week (Saturday) compared to the beginning (Monday *p* = 0.01; Table 3).

## Discussion

The present study describes the heat stress and kidney strain daily experienced by Mesoamerican sugarcane cutters. Throughout a weekly work–rest cycle, biomarker dynamics for kidney glomerular filtration, muscle injury and systemic inflammation were marked and illustrate the daily physical stress, the subsequent physiological strain, and recovery experienced by workers. The degree of physiological strain experienced by workers in the current study occurred despite workers experiencing a lower level of heat strain due to lower levels of environmental heat stress than that typically experienced over the harvest plus the implementation of a structured and monitored Rest.Shade.Hydration workplace heat stress intervention (Glaser et al. 2022).

### Context during and away from work

Workload intensity was the highest on Monday in the current study, raising the question of whether a longer recovery period (42 h) allowed workers to maintain a higher work intensity on Monday or if the higher daily average WBGT recorded on Monday created a more stressful work environment and consequently a greater physical and cardiorespiratory strain. Individual-level production data were not collected, but company records indicate that burned cane cutters generally cut approximately 11% and 16% more on Mondays than Fridays or Saturdays respectively, strongly suggesting that workers are better rested after a weekend and therefore maintain higher production rates at the start of their work week.

Neighborhood characteristics (e.g., rural vs. urban; residential greenness) and residential structures and infrastructure (e.g., roofing material, power supply, cooling devices) strongly influence heat exposure, heat stress and mortality. Yet, most epidemiological studies rely on metrological data from local weather stations that may not accurately reflect a populations’ 24 h heat exposure (Tasgaonkar et al. 2022). To our knowledge, this is the first study to examine sleep and physical activity away from work in sugarcane workers. Notably, workers slept for approximately 5 h the night before work, with ambient temperature within their homes ranging between 23 and 35 °C while they slept. Nighttime air temperatures reported in the current study were at the upper limit of thermal comfort levels, where heat-related sleep disruptions can occur (Tsuzuki et al. 2017). Furthermore, on average, workers had to wake at 02:40 to travel and start work at dawn (approx. 05:30). An increased risk of CKD development (Park et al. 2020) and progression (Chen et al. 2022b) has been associated with short sleep duration (≤ 6 h) and poor sleep quality. Therefore, further work on the daily routines and daily living practices in this at-risk population is needed to further characterize potential health risk factors.

### Work–recovery cycle: kidney, hydration and muscle damage biomarker dynamics

The current study showed cross-shift changes in serum creatinine similar to those previously reported in heat-stressed sugarcane cutters/workers (García-Trabanino et al. 2015; Santos et al. 2015; Butler-Dawson et al. 2019; Wegman et al. 2018); all markedly different from the expected lack of diurnal variation in creatinine seen in a temperate environment with light exercise allowed (Sennels et al. 2012). A novel finding from this study was that recovery of cross-shift changes in kidney strain and muscle injury biomarkers were similar after one night (18 h) as compared to two nights (42 h) of rest. No study to date has examined kidney glomerular function short-term recovery rates in workers at risk of AKI and CKDnt. Studies have repeatedly shown a high rate of decline in kidney function across a 5–6 month harvest period (Kupferman et al. 2018; Hansson et al. 2019b; Wegman et al. 2018; Wesseling et al. 2016). A more marked eGFR reduction in workers experiencing larger cross-shift kidney marker changes has also been reported (Butler-Dawson et al. 2019; Santos et al. 2015; García-Trabanino et al. 2015; Sorensen et al. 2019; Dally et al. 2020). Interestingly, one previous study in Nicaraguan sugarcane cutters (Wesseling et al. 2016) found a less-pronounced cross-shift increase in serum creatinine as compared to other studies in similar populations (Wegman et al. 2018; García-Trabanino et al. 2015; Santos et al. 2015), with the authors attributing this to post-shift samples being taken in the evening several hours after work ended (i.e., after some period of recovery).

Previous laboratory-based studies have shown that markers of acute kidney injury/strain return to baseline values within 24 h in healthy, unacclimated participants performing a bout of prolonged exercise in the heat (Chapman et al. 2019, 2020). In the current study, workers were heat-acclimatized and recovered their kidney function in under 24 h, despite having performed strenuous work in a hot environment for five consecutive days prior, and three months into the harvest season. Therefore, pre-shift-to-pre-shift changes are likely indicative of an actual decline in kidney function, as reported by Dally et al. (2020) where worsening pre-shift serum creatinine levels over a work week forecasted end-of-harvest kidney function in agricultural workers. The current study, together with preceding studies (Wesseling et al. 2016; Hansson et al. 2022), highlights the need for appropriate timing of sample collection when examining kidney function, especially in physically active and/or heat-stressed populations. For assessment of kidney function in manual workers, sampling in the morning/pre-shift is essential. This study indicates that pre-shift samples taken the morning after a work shift can be used to reflect steady state kidney function (Fig. 1).

This is the first study to report changes in cystatin C across sugarcane cutter work shifts. Cystatin C is synthesized from all nucleated cells at a constant rate that is diet-, muscle- and physical activity-independent (Chen et al. 2022a). Cross-shift changes in cystatin C were markedly different from diurnal changes previously observed across 24 h in a controlled environment (Sennels et al. 2012). Interestingly, cross-shift increases in serum cystatin C were more pronounced than serum creatinine increases, indicating that decreases in eGFR_crea_ during a work shift are not merely due to muscle release of creatinine during physical exertion. In agreement with this, Andersson et al. (2022) reported that cross-harvest eGFR_cysc_ changes were well correlated with eGFR_crea_ changes in 458 workers from the same workplace. Although eGFR_crea_ was found to be a better marker than eGFR_cysc_ for GFR measured using iohexol clearance in a Mesoamerican population (Raines et al. 2023a), the more evident cross-shift increase in cystatin C demonstrated in the current study, and Andersson’s previous findings indicate that cystatin C is a valuable cross-shift and cross-harvest marker of kidney strain in sugarcane workers, especially as it is independent of muscle catabolism.

In this study, we do not ascribe post-shift increases in serum cystatin C or serum creatinine to dehydration-induced hemoconcentration since workers’ measured fluid consumption was on average 8 L at work and their hydration biomarkers indicate they were adequately hydrated both before and after their work shifts. Rather than dehydration, it is more likely that post-shift increases in glomerular filtration markers were due to exertional heat stress-induced reductions in renal blood flow. Indeed, thermoregulatory demands and blood redistribution significantly reduce renal blood flow (Radigan and Robinson 1949). These renal circulatory changes concur with dynamics in circulating levels of muscle injury and systemic inflammation biomarkers, as indicated by the current and previous studies (Wegman et al. 2018; Santos et al. 2015), and may be relevant for the development of CKDnt (Hansson et al. 2020). Workers’ serum sodium levels in the current study remained mostly stable across the work shift, with four out of 60 post-shift samples indicating mild hyponatremia (134–136 mmol/L). In a previous study using an intervention with a strong hydration focus, Guatemalan sugarcane workers drank on average 18–19 L of water and electrolyte beverages per day (Krisher et al. 2020; Sorensen et al. 2019). The mean post-shift sodium and potassium levels were at the lower reference interval limits (135 mmol/L and 3.5 mmol/L, respectively) in workers with a mean water intake of 15 L over the shift (Sorensen 2019). Another study showed post-shift asymptomatic hyponatremia; < 135 mmol/L was common (19–28%) with up to 20 L fluid intake over a 3 week period (Krisher et al. 2020). Acute health risks exist for both inadequate (e.g., increased physiological strain) and excessive (e.g., hyponatremia) water intake (Cotter et al. 2014). Workers should have easy access to rehydration fluids and receive training to maximize the benefit of rehydration while minimizing the risk of hyperhydration. Easy access to water for skin cooling purposes should also be advocated.

Consistent with a previous study among Salvadoran sugarcane cutters (Wegman et al. 2018), the current study found CPK levels increased during the work shift, implying muscle damage or strain. Larger cross-shift changes in CPK levels have been reported in sugarcane workers who have suffered an acute kidney injury (Santos et al. 2015). Thus, episodes of muscle injury that lead to myoglobinuria and kidney inflammation remain a potential driver of AKI and CKD (Candela et al. 2020). In the current study CRP, an indicator of systemic inflammation was generally higher at the end of the working week than in the beginning, consistent with accumulating low-grade inflammation during the working week, followed by (at least partial) resolution over the weekend. Such occupationally induced inflammation has been hypothesized to be a contributing cause of CKDnt (Hansson et al. 2020). As CRP levels peak 24–48 h after inflammatory stimuli (Schmit and Vincent 2008), it was not considered meaningful to analyze cross-shift changes. To explore whether hard work in heat can be linked to systemic inflammation over this time frame, future studies should consider more rapidly responding markers of systemic inflammation, such as neutrophil-to-lymphocyte ratio, which is considered a potential marker of overtraining (Walzik et al. 2021), and explore the role which increased gut permeability may have in fueling such inflammation (Houser et al. 2021; Raines et al. 2023b).

Serum potassium has a marked diurnal variation, with the levels peaking before noon and the lowest level in the afternoon/evening (13:00 – 00:00) (Sennels et al. 2012). Moreover, although potassium concentration increases during physical exercise, upon cessation of exercise, potassium concentration rapidly decreases (within 30 s) as potassium again enters the skeletal muscle, often falling to below resting values within 5 min of exercise ending (Lindinger 1995). These mechanisms may well explain the lower serum potassium level post-shift in the current study. It should be noted that workers consumed on average four 300 mL *bolis* each work shift, which likely helped workers to maintain electrolyte homeostasis.

### Considerations

This study was not designed to address the implications of repeated cross-shift changes and limited recovery on kidney function/health over a longer timeframe, such as a harvest. A limitation is that we did not include a comparison population undertaking less physically demanding work but exposed to the same heat in the field. In addition, this study was conducted in a population receiving a robust Rest.Shade.Hydration intervention and during work shifts where environmental conditions were at the lower range of what is common during a harvest period. The work–recovery cycle among groups working with insufficient occupational interventions requires investigation. It is possible that our observation of workers may have altered their behavior (e.g., workers’ self-pacing strategy during work or their fluid intake). Telemetric pill ingestion ~ 1 h prior to workers starting their shift likely did not influence the validity of our T_GI_ measures (Notley et al. 2021). However, water ingestion during the work shift appeared to, which agrees with what others have shown (Wilkinson et al. 2008). Therefore, T_GI_ measures may underestimate the degree of heat strain experienced by workers in the current study.

## Conclusion

Findings from this and other studies clearly call for adherence to well-timed sampling of biological samples when investigating kidney health in occupational populations. Sampling should be performed in the morning, before the start of work, to reflect baseline kidney function. However, the day of the working week, at least in a population receiving an intervention, seems not to influence the results.

The conceptual framework (Fig. 1) provides a guide for selecting measurable outcomes for health surveillance (with a focus on kidney health) in working populations. Measures of heat stress and kidney strain outlined in this model are immediate outcomes that are likely to respond rapidly and directly reflect changes in work practices.

Our results show that despite implementation of an advanced Rest.Shade.Hydration workplace intervention, sugarcane cutters routinely present with changes in cystatin C, creatinine, CPK and CRP that are indicative of daily kidney and muscle strain, and possibly accumulation of systemic inflammation across the work week. Although these cross-shift biochemical fluctuations seem to resolve by the start of the next workday, at least in this population with RSH, they highlight the physiological strain experienced by these workers day by day and week by week. The long-term impacts of such repeated bouts of kidney strain require further investigation. Continued surveillance in workforces repeatedly exposed to occupational heat stress is needed as are continued efforts to improve workplace heat prevention and implement protective interventions in industrial agriculture and other industries.

## Supplementary Information

Below is the link to the electronic supplementary material.

Supplementary file1 (DOCX 32 KB)

## Data Availability

The data supporting the findings of this study are available within the article and its supplementary materials. Further inquiries can be directed to the corresponding author.

## Abbreviations

CKDnt: Chronic kidney disease of non-traditional origin
CRP: C-reactive protein
ISA: Ingenio San Antonio
IQR: Interquartile range
CKD-EPI: Chronic kidney disease epidemiology collaboration
T_GI_: Gastrointestinal temperature
%HRmax: Percentage age predicted maximal heart rate
CPK: Creatine phosphokinase
ANOVA: Analysis of variance
CI: Confidence intervals
eGFR: Estimated glomerular filtration rate
WBGT: Wet-bulb globe temperature
eGFR_crea_: Estimated glomerular filtration rate for serum creatinine
eGFR_cysc_: Estimated glomerular filtration rate for serum cystatin C
