# Supplementary material for: The work–recovery cycle of kidney strain and inflammation in sugarcane workers following repeat heat exposure at work and at home
Source: Eur J Appl Physiol. 2024 Oct 5;125(3):639–52. doi: 10.1007/s00421-024-05610-3 (PMC11889006; doi:10.1007/s00421-024-05610-3)
Supplement: Supplementary file 1 — Supplementary file1 (DOCX 32 KB) [file 421_2024_5610_MOESM1_ESM.docx]

**Supplementary material**

**Supplementary Table 1:** Kidney function (CKD-EPI eGFR) in burned sugarcane cutters post-shift to pre-shift after one night’s recovery (Friday post-shift to Saturday pre-shift, 18-h off work) and two night’s recovery (Saturday post-shift to Monday pre-shift, 42-h off work).

*EPI denotes eGFR is estimated according to the Chronic Kidney Disease Epidemiology Collaboration equation (Levey et al 2009).*

| Biomarker | Concentrations, median (IQR) | | | | | Concentration changes, median (IQR) | | | | p-values | | |
| --- | --- | --- | --- | --- | --- | --- | --- | --- | --- | --- | --- | --- |
|  |  |  |  |  |  |  |  |  |  |  |  |  |
|  | Friday PM | Saturday AM | Saturday PM | Monday AM | Monday PM | Friday-Saturday recovery (18h) | Saturday cross-shift (post 18h recovery) | Saturday-Monday recovery (42h) | Monday cross-shift (post 42h recovery) | cross-shift v.s. recovery effect | different recovery effect 18h vs 42h | different cross-shift effect after 18h vs. 42h recovery |
| eGFR_cys C_ (ml/min/1.73m^2^) | 91 (75-108) | 101  (89-113) | 84  (72-99) | 95  (88-104) | 81  (67-93) | 1.07 (1.03-1.17) | 0.89 (0.85-0.95) | 1.08 (1.05-1.15) | 0.85 (0.80-0.91) | <0.001 | 0.70 | 0.10 |
| eGFR_crea_ (ml/min/1.73m^2^) | 111 (91-121) | 117 (103-123) | 107  (92-120) | 115 (90-123) | 101  (92 -121) | 1.06 (0.98-1.13) | 0.95 (0.89-0.98) | 1.04 (0.95-1.13) | 0.93 (0.87-0.97) | 0.02 | 0.19 | 0.71 |

**Supplementary Table 2:** Daily average and maximum Wet Bulb Globe Temperature (WBGT), dry bulb temperature, globe temperature and relative humidity across three burned cane cutting workdays.

|  | WBGT (°C) | | Dry bulb temperature (°C) | | Globe temperature (°C) | | Relative Humidity (%) | |
| --- | --- | --- | --- | --- | --- | --- | --- | --- |
|  | Average | Max | Average | Max | Average | Max | Average | Max |
| Friday | 26.9 | 27.7 | 32.1 | 34.5 | 40.0 | 42.6 | 32 | 42 |
| Saturday | 27.7 | 29.3 | 32.9 | 36.3 | 42.9 | 46.3 | 27 | 31 |
| Monday | 29.0 | 30.8 | 32.1 | 33.6 | 44.4 | 49.3 | 32 | 37 |
